# Competing rhythmic neural representations of orientations during concurrent attention to multiple orientation features

**DOI:** 10.1038/s41467-019-13282-3

**Published:** 2019-11-20

**Authors:** Ce Mo, Junshi Lu, Bichan Wu, Jianrong Jia, Huan Luo, Fang Fang

**Affiliations:** 1 Center for Studies of Psychological Application, School of Psychology, South China Normal University, Guangzhou, China; 2 School of Psychological and Cognitive Sciences and Beijing Key Laboratory of Behavior and Mental Health, Peking University, Beijing, China; 3 IDG/McGovern Institute for Brain Research, Peking University, Beijing, China; 4 Peking-Tsinghua Center for Life Sciences, Peking University, Beijing, China; 5 Institut des Sciences Cognitives Marc Jeannerod, Centre National de la Recherche Scientifique, UMR 5229, Bron, France

**Keywords:** Attention, Perception

## Abstract

When a feature is attended, all locations containing this feature are enhanced throughout the visual field. However, how the brain concurrently attends to multiple features remains unknown and cannot be easily deduced from classical attention theories. Here, we recorded human magnetoencephalography signals when subjects concurrently attended to two spatially overlapping orientations. A time-resolved multivariate inverted encoding model was employed to track the ongoing temporal courses of the neural representations of the attended orientations. We show that the two orientation representations alternate with each other and undergo a theta-band (~4 Hz) rhythmic fluctuation over time. Similar temporal profiles are also revealed in the orientation discrimination performance. Computational modeling suggests a tuning competition process between the two neuronal populations that are selectively tuned to one of the attended orientations. Taken together, our findings reveal for the first time a rhythm-based, time-multiplexing neural machinery underlying concurrent multi-feature attention.

## Abstract (web summary)

The neural mechanisms for concurrently attending to multiple features in the visual stimuli are not well understood. Here, the authors show that the neural representations for two overlapping stimulus features alternate with each other at a ~4 Hz rhythm that was also observed in fluctuations in the task performance.

## Introduction

Attention, in addition to being able to dwell on certain locations or objects, can also be selectively tuned to particular features that are distributed over multiple spatial locations or objects in a visual display (i.e., feature-based attention)^1,2^. It was found that this process is mediated by a feature similarity gain mechanism^3,4^ that operates in all neurons regardless of their receptive field locations, thereby enhancing the representation of the attended feature(s) throughout the whole visual field^5,6^. However, an equally and even more important, but less recognized question is how the visual system attends to multiple (more than one) features and how the neural representations of the features are coordinated with each other in the brain. Neurons encoding a feature (e.g., orientation) could be found throughout visual cortex and are even more dispersed than those encoding a spatial location^7–9^. As a consequence, attention to multiple features might require a distinctive neural mechanism that globally coordinates activities among multiple dispersed neuronal populations that tuned to the attended features, which remains largely unknown despite recent behavioral evidence for rhythmicity in feature-based attention^10^.

The key to addressing this question is to investigate how the neural representation of each attended feature is modulated during multi-feature attention. A growing number of studies have shown that attention, instead of being stationary, is a highly dynamic and flexible process that organizes a multitude of spatial or object representations in the temporal dimension^11–15^. For example, attention samples targets (e.g., locations, objects) rhythmically, with different targets being processed in different phases^16,17^. These findings implicate that multi-feature attention might also rely on a temporal coordination process in which attention resources are dynamically allocated between multiple features over time. Understanding the neural mechanism of such a process hence necessitates characterizing its temporal dynamics, which is nonetheless concealed to time-insensitive approaches.

Here we recorded magnetoencephalography (MEG) signals from human subjects while they concurrently attended to two orientation features and performed an orientation discrimination task similar to Herrmann et al.^18^. A multivariate inverted encoding model (IEM)^19–22^ was applied to the MEG signals to reconstruct the neural representations of these two features at each time point throughout the attentional process. Benefiting from the high temporal resolution of MEG recordings, the moment-to-moment reconstruction results allowed us to assess the fine temporal courses of the orientation representations during multi-feature attention. We found that the representations of the two concurrently attended orientations alternated with each other as they underwent a theta-band (~4 Hz) rhythmic fluctuation process, showing an anti-phase temporal relationship. Furthermore, we performed a time-resolved behavioral study using the same multi-feature attention task, and the results showed a similar rhythmic profile as that in the MEG signals, supporting an essential link between the neural and behavioral findings. Finally, the temporal rhythmicity in the orientation representations was associated with a competition process between the neuronal populations tuned to the attended orientations, manifested as periodical changes in their tuning widths.

## Results

### Time-resolved representation of single orientation feature

**Fig. 1:**
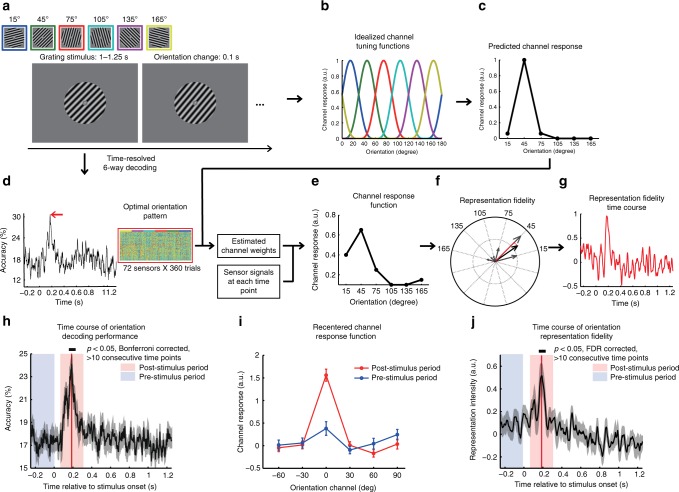
IEM-based time-resolved reconstruction of orientation representation. **a** Stimuli and trial structure in the model training part. Subjects performed an orientation discrimination task at one of six orientations (15°–165° in steps of 30°, coded in different colors) that corresponded to the preferred orientation of six orientation channels. **b** The idealized tuning of these channels were characterized as smooth, Gaussian-like functions centered at their respective preferred orientation and plotted in the corresponding colors as in **a**, which spanned the entire orientation space. **c** Channel responses to a given orientation (e.g., 45°) could thus be predicted based on the functions. **d** The time-resolved decoding analysis was performed on the model training part data. The pattern of instantaneous MEG sensor signals that achieved the highest decoding accuracy (indicated by the red arrow) was extracted as the optimal orientation pattern (shown inset). Correspondence of trial-wise vectors of sensor signals (organized in columns) to the six orientations was color coded in the top row. The contribution of each orientation channel (i.e., weight) to the MEG signal from each sensor was estimated based on the optimal orientation pattern. These estimated weights were then inverted and applied to each time point to reconstruct the instantaneous channel response function at that time point, as shown in **e**. **f** The degree to which the reconstructed channel responses encoded the information of an orientation (e.g., 45°) was quantified as representation fidelity, which was calculated as the projection of the mean vector onto the direction corresponding to that orientation. **g** The reconstruction procedure was performed at each time point, yielding a continuous representation fidelity time course. **h** Time course of orientation decoding performance. **i** Averaged channel response functions across the six orientations with the reference channel (0°) at their respective peak orientation. Error bars denote one SEM across subjects. **j** Time course of orientation representation fidelity averaged across the six orientations. The gray-shaded area around the curve denotes one SEM across subjects and the red solid line indicates the time point of the highest decoding performance in **h** and **j**

The MEG experiment consisted of two independent parts: the model training part and the attention part. IEM hypothesized that MEG signals in each sensor could be modeled as a weighted sum of the responses of six orientation channels that are selectively tuned to different orientations (Fig. 1a–c). These weights were first estimated based on the data in the model training part, and then were used to reconstruct the orientation channel responses in the attention part when subjects concurrently attended to two orientations. Importantly, the reconstruction was performed on the MEG signals at each time point, and we could thus assess the time-resolved orientation representations during multi-feature attention (Fig. 1d–g). It is noteworthy that the model training and reconstruction were performed for individual subjects.

In the model training part, subjects were presented with a single sinusoidal grating stimulus with one of six orientations and performed an orientation discrimination task (Fig. 1a). At each time point, we used a support vector machine classifier to classify the trial-wise vectors comprised of sensor signals into one of six orientation categories (Fig. 1d). We found that, at the group level, orientation information could be reliably decoded from MEG signals between 167 and 215 ms (one-sample *t*-test: *p* *<* 0.05, Bonferroni corrected for more than 10 consecutive significant time points), with the highest decoding accuracy at 180 ms (Fig. 1h). This temporal profile was consistent with previous studies^23,24^. We obtained the optimal orientation pattern as the trial-wise vectors of sensor signals at the time point with the highest decoding accuracy for the IEM training (i.e., weight estimation), as they presumably conveyed the richest orientation information. The time-resolved orientation channel responses could then be reconstructed from the MEG signals in both the model training part and the attention part.

We first validated the trained IEM based on the model training data by examining the channel response functions in the pre-stimulus period (−250 to 0 ms, blue shade) and the post-stimulus period (250 ms time window centered at the time point with the highest decoding accuracy for each subject, red shade, Fig. 1j). For both periods, a channel response function was calculated for each of the six presented orientations by averaging the vector of channel responses across all time points. The peak orientations of these channel response functions thus corresponded to each of the six orientations. These channel response functions were circularly shifted to align their respective peak orientations to a common 0° center such that they could be averaged across the six orientations (re-centering). It should be noted that the re-centering should not produce any significant 0° peak in the absence of orientation information. As shown in Fig. 1i, the averaged channel response function showed a clear bell-like profile with the highest response at the 0^°^ center for the post-stimulus period (red line, one-way ANOVA: *F*_(5,84)_ = 49.59, *p* *<* 10^−9^) but not for the pre-stimulus period (blue line, *F*_(5,84)_ = 2.11, *p* *>* 0.05). This indicates that the orientation information was strongly represented in the post-stimulus period but not in the pre-stimulus period.

We then examined whether the trained IEM could capture orientation information changes in the MEG signals at a high temporal resolution, by computing the representation fidelity metric^21^ of the presented orientation at each time point of the model training data. At the group level, we observed reliable above-zero orientation representation fidelity (one-sample *t*-test, *p* *<* 0.05, FDR corrected for more than ten consecutive significant time points) from 172 to 220 ms, with the highest representation fidelity at 187 ms. This was well consistent with the time course of the decoding performance. Thus, the IEM trained by the optimal orientation pattern is reliable for reconstructing the fine temporal dynamics of orientation representation from the MEG signals, at least when only one orientation feature was present.

We performed two control analyses to further validate our IEM approach. First, to examine the model performance on random data, we scrambled the orientation labels of the instantaneous trial-wise sensor signals at each time point and performed the same IEM analyses on the random surrogate data. We found that the re-centered channel response function in the post-stimulus time period exhibited a flat profile (*F*_(5,84)_ = 0.26, *p* *=* 0.93, Supplementary Fig. 2a) with little representation of orientation information. Second, to investigate how the selection of the optimal orientation pattern by the SVM classifier might affect the outcome, we selected the surrogate optimal pattern as the instantaneous trial-wise vectors of sensor signals at the time point of the highest SVM decoding performance in the pre-stimulus period (baseline data), and performed the same IEM analysis. If our findings were somehow contingent on our approach of determining the optimal orientation pattern, rather than the orientation information itself, then one might expect a similar bell-shaped profile in the channel response function obtained from the surrogate optimal pattern. However, we did not observe such a profile in the channel response function for the post-stimulus period (*F*_(5,84)_ = 0.59, *p* *=* 0.71, Supplementary Fig. 2b). Together, these results suggest that our findings are unlikely to arise by chance and could not be explained by the approach of selecting the optimal orientation pattern.

### Neural representation of multiple attended orientations

**Fig. 2:**
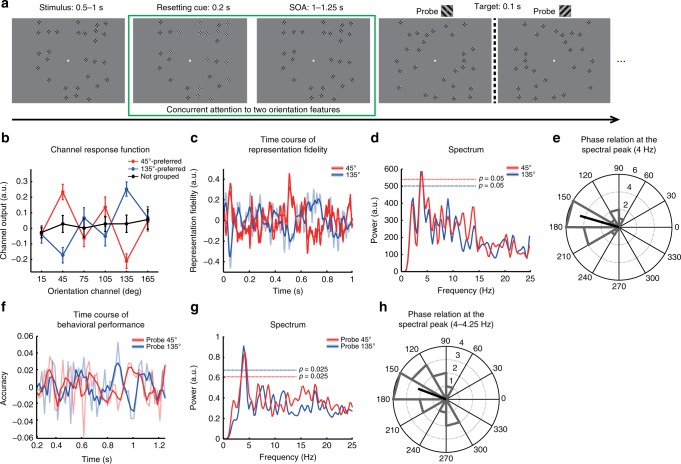
Rhythmic fluctuation of the orientation representations. **a** Trial structure in the attention part. **b**–**e** MEG results in the attention part. **b** Mean channel response functions with the preferred channel being the 45° channel (red) and 135° channel (blue), respectively. Error bars denote one SEM across subjects. **c** Representation fidelity of the two attended orientations as a function of time relative to the resetting cue (zero point). For better visualization, the smoothed time courses (in saturated colors) are overlaid on the unsmoothed time courses (in desaturated colors). **d** Spectra of the representation fidelity time courses for the two attended orientations. Dashed lines denote the statistical thresholds (corrected for multiple comparisons). **e** Distribution of the phase difference between the two orientation representation fidelity time courses at the spectral peak (4 Hz) across subjects. The thick black bar indicates the mean of the phase differences. **f**–**h** Time-resolved psychophysical results. **f** Orientation discrimination performance as a function of time. **g** Spectra of the behavioral performance time courses for the two attended orientations. **h** Distribution of the phase difference between the two behavioral time courses at the spectral peaks (4–4.25 Hz) across subjects. The thick black bar indicates the mean of the phase differences

Having validated the trained IEM based on the model training part data, we then used the IEM to reconstruct the time courses of orientation representations in the attention part when subjects concurrently attended to two orientation features (green box, Fig. 2a). In the attention part, subjects were presented with a multitude of sinusoidal gratings dispersed randomly. Half of the gratings were oriented at 45° while the others were oriented at 135°. An abrupt contrast increase with all the gratings of one orientation was introduced in each trial to presumably reset the attentional process^13,25^. So that we could examine the moment-by-moment neural representations of the orientation features with reference to a common temporal marker. Subjects were required to report a near-threshold orientation change (i.e., clockwise or anticlockwise) in the upcoming probe with respect to its closest orientation in the initial stimulus (Fig. 2a).

First, we examined whether the reconstruction results contained the information about the two attended orientations (45^°^ and 135^°^). We calculated the channel response function for the 1000 ms post-cue period, by averaging the vectors of instantaneous channel responses across time points. Surprisingly, the channel response function showed a relatively flat profile for all the orientations with no elevated responses in either the 45° channel or the 135° channel (black line, Fig. 2b, one-way ANOVA: *F*_(5,84)_ = 0.29, *p* *=* 0.9). A possible reason is that the competition between the two attended features resulted in cancelation of channel responses in the time-averaged results. To test this possibility, we grouped the vectors of instantaneous channel responses into two sets based on whether the response was higher in the 45° channel than in the 135° channel (45°-preferred) or vice versa (135°-preferred), regardless of the responses in the other channels. We then calculated the channel response function separately for each set. Interestingly, both channel response functions exhibited a clear peak at their preferred channel (paired *t*-test: 45°-preferred: *t*_(14)_ = 3.01, *p* *<* 0.01; 135°-preferred: *t*_(14)_ = 3.64, *p* *<* 0.01), as well as a clear trough at their non-preferred channel (e.g., the 135° channel for the 45°-preferred function) in comparison to the other channels (paired *t*-test: 45°-preferred: *t*_(14)_ = −3.4, *p* *<* 0.01; 135°-preferred: *t*_(14)_ = −2.66, *p* *<* 0.025, Fig. 2b). Thus, these results indicated that the information about both the two attended orientations were maintained in the MEG signals. More importantly, the representations of the two orientations might be engaged in a competition in which they dominated over each other at different moments by simultaneously enhancing one and suppressing the other.

### θ-band rhythmic multi-orientation representations

After revealing the possible competing relationship between the two orientation features, we further assessed the fine temporal characteristics of the two orientation representations. We calculated the representation fidelity metric for each of the attended orientations respectively at each time point throughout the 1000 ms temporal interval. As shown in Fig. 2c, both moment-to-moment orientation representations (red: 45°; blue: 135°) showed a rhythmic fluctuating pattern. Moreover, the two orientation representations seemed to exhibit an alternating relationship such that the peaks of one coincided with the troughs of the other, and vice versa. Indeed, this time-resolved pattern was consistent with the flat time-averaged result (Fig. 2b). To examine the spectral contents of the orientation representation time courses, we performed a spectral analysis on the orientation representation fidelity time courses averaged across trials for each subject. The amplitude spectra were then averaged across subjects for each of the two orientations. As shown in Fig. 2d, both orientations showed a significant peak in the theta band (permutation test, corrected for multiple comparisons, *p* *<* 0.05; 45°: 3.75–4 Hz; 135°: 3.5–4 Hz). Thus, the temporal fluctuations of the two orientation representations were not stochastic but displayed a theta-band rhythmical pattern.

To further test the alternating relationship between the time courses of the two orientation representations, we calculated their phase difference in the theta-band for each subject. As shown Fig. 2e, the 45°–135° phase difference was not uniformly distributed across subjects, but was clustered around 164° (Rayleigh test for uniformity, *p* *<* 0.001) that was significantly different from 0° (Rayleigh test, *p* *<* 0.001) but not significantly different from 180° (Rayleigh test, *p* *=* 0.16), supporting their anti-phase relationship in the theta band. Therefore, the neural representations of the two concurrently attended orientations followed a rhythmically oscillating trajectory in which the enhancement of one feature representation was accompanied by the suppression of the other.

### Behavioral oscillation in multi-orientation attention

Previous studies, by employing a time-resolved behavioral approach, have demonstrated neurophysiologically relevant rhythms in behavior^10,11,13,14^. If multi-feature attention is indeed mediated by a rhythmic sampling neural mechanism, as suggested by our MEG results, we would expect a similar rhythmic profile in the time-resolved behavioral performance using the same multi-feature attention paradigm. We therefore employed the same stimuli and experimental procedure as those in the attention part of the MEG experiment, in combination with time-resolved psychophysics, to assess the temporal dynamics of multi-feature attentional process at the behavioral level. The only modification was the introduction of a systematically varied temporal lag between the cue and the probe (SOA), such that the probe appeared at one of 50 SOAs (50–1050 ms in steps of 20 ms) with equal probability.

Figure 2f illustrates the orientation discrimination accuracy as a function of cue-to-probe SOA for the 45° (red) and the 135° (blue) orientations, respectively. The behavioral results showed a fluctuating temporal profile as well as an alternation between the two orientations. We then applied the same statistical procedure as that used for the MEG data to the time-resolved behavioral data, and identified a significant theta-band spectral peak (permutation test, corrected for multiple comparisons; 45°: 3.75–4.5 Hz, *p* *<* 0.025; 135°: 3.75–4.25 Hz, *p* *<* 0.025) for both orientations (Fig. 2g). Moreover, sensitivity to the two orientation features exhibited an anti-phase relationship in the theta band (Rayleigh test for uniformity, *p* *<* 0.01), and the distribution of the phase difference across subjects significantly deviated from 0° (Rayleigh test*, p* *<* 0.01) yet did not differ from 180° (Rayleigh test*, p* *=* 0.23) (Fig. 2h). Notably, even at the single subject level, the individual behavioral time courses exhibited a clear anti-phase relationship between the two conditions (Supplementary Fig. 1a), which was consistent with the group results. Moreover, the individual power spectra were also highly consistent across subjects, with a theta-band spectral peak for both the probe 45° (red) and the probe 135° (blue) conditions (Supplementary Fig. 1b). Thus, the behavioral findings further advocated that the two attended orientation features were sampled in a rhythmic and alternating manner.

### Tuning shift model vs. tuning competition model

**Fig. 3:**
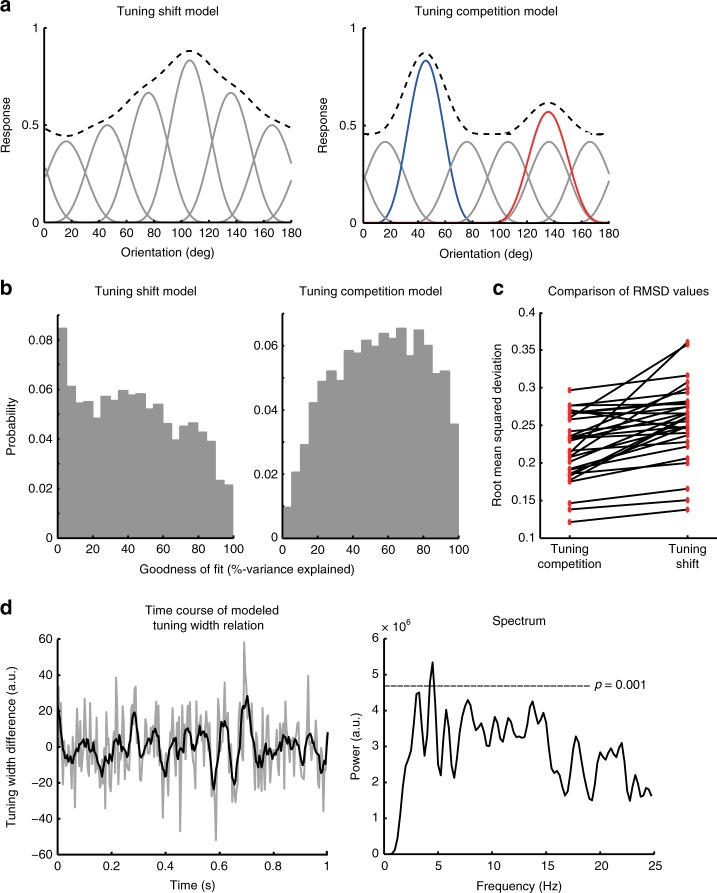
Results of computational modeling. **a** Illustration of the tuning shift (TS) model (Left) and the tuning competition (TC) model (Right). The TS model postulates that attention shifts the tuning of individual neurons (gray curves) towards the attended feature, which results in a uni-modal population response profile (black dashed curve) centered at the attended feature. In particular, when two features are attended, individual neuronal tunings would shift periodically between these two features in a similar manner, thus leading to a rhythmic oscillation of the population response profile center. In contrast, the TC model postulates an ongoing competition between the neuronal populations tuned to the attended features (red and blue curves) in multi-feature attention while the other neurons remain little affected. This leads to a multi-modal population response profile with local peaks at the attended features that rhythmically alternate in dominance. **b**, **c** Model comparison results. **b** Goodness-of-fit (GoF) distributions across all time points pooled from all subjects. **c** RMSD values averaged across all time points plotted as paired red dots for individual subjects. **d** The modeled tuning width difference between the two neuronal populations plotted as a function of time (left) and its spectrum (right). The smoothed time course (in black) is overlaid on the unsmoothed time course (in gray). The gray dashed line denotes the statistical threshold for the spectrum (corrected for multiple comparisons)

Both MEG and behavioral results have demonstrated a theta-band rhythmic oscillation in multi-feature attention, yet it remains unclear what type of neural mechanism could account for these observations. One possibility is that the periodic enhancement of the feature representation is associated with the tuning shifts of individual neurons towards the currently sampled feature, as in the uni-feature attention^8,26^. The tuning shifts would result in a uni-modal population response profile (black dashed curve, Fig. 3a, left) whose peak periodically shifts between the two attended orientations at theta band (tuning shift (TS) model). An alternative possibility is that the rhythmic sampling is related to the competition between the two neuronal populations that are selectively tuned to the attended features (red and blue curves, Fig. 3a, right). In this case, the ongoing neuronal competition would lead to a bi-modal population response profile with two local peaks that periodically alternate in dominance at theta band (tuning competition (TC) model).

To distinguish between these two possibilities, we fitted both the TS and TC models to the population response profiles (sum of idealized channel tuning functions weighted by their respective response) and measured their goodness of fit (GoF) at each time point. The GoF distribution of the TC model was more positively skewed (median = 0.6) in comparison to the TS model (median = 0.43), and the TS model fitted poorly (GoF < 0.1) for approximately 9% of all the time points (Fig. 3b), suggesting that the TC model showed an overall better fit than the TS model. To further compare the two models, we evaluated the model fitness by calculating the root mean squared deviation (RMSD) metric^27^. RMSD takes the number of model parameters into account, and a smaller RMSD indicates better model fitness. As shown in Fig. 3c, the results again advocated the TC model over the TS model (Wilcoxon’s signed rank test, *p* *<* 0.001). Interestingly, we also identified a theta-band oscillatory pattern for the estimated moment-to-moment tuning width difference between the two neuronal populations (permutation test, multiple comparisons corrected, 4.25–4.5 Hz, *p* *<* 0.001, Fig. 3d) but not for the amplitude difference, indicating that the competition between the two neuronal populations mainly involved tuning width changes. Together, these results suggested that the rhythmicity in multiple-feature attention was likely to arise from the competition between the neuronal populations tuned to the attended orientations, rather than global tuning shifts of all orientation selective neuronal populations.

## Discussion

We employed a time-resolved multivariate IEM approach on MEG data to track the time courses of ongoing neural representations of two concurrently attended orientations. We found that the neural representations of the two orientations, instead of being simultaneously and persistently enhanced, waxed and waned in a theta-band rhythm and alternated with each other over time. This temporal pattern was also observed in the time-resolved performance of an orientation discrimination task when subjects concurrently attended to the two orientations. Using computational modeling, we found that this pattern might be mediated by a tuning competition process, whereby neuronal groups tuned to one of the attended orientations modulate their tuning width at different phases of a theta-band cycle. Together, our findings support a rhythmical attention shift in the feature space, suggesting that the brain might employ a time-multiplexing approach to represent and maintain multiple attended features.

The temporal dynamics of orientation representation we discovered here suggest that attention resource could be flexibly switched and reallocated among multiple features over time. Consistent with a very recent behavioral study that revealed rhythmic structures in the time-resolved behavioral performance of a feature-based attention task^10^, our findings could be conceptualized as the attended orientations being alternatively lightened by a feature-based attention spotlight that bypasses spatial topography. The notion of an attention spotlight scanning through the feature space is consistent with an earlier finding that observers could successfully track visual objects that continuously changed in the feature space^28^. Notably, our findings bear directly on the fundamental question whether spatial and feature-based attention are mediated by similar neural mechanisms^2,29–31^. Neurophysiological studies have found that both firing rates and spike correlations between neuron pairs are similarly affected by the two forms of attention. Specifically, attending to a location or a feature increases the response of the neurons whose preferred feature or location matches that of the attention target^2,32,33^ and simultaneously decreases their response correlations^7^. Our data are in line with these findings. At least for multiple attention targets, the two attention forms operate at the same time scale (i.e., ~4 Hz), thus suggesting similar mechanisms mediating the two attention forms.

Our findings, from both MEG recording and behavioral measurement, consistently show that attention to multiple features is coordinated at a theta-band rhythm, a rhythm known to implement attentional allocation among multiple locations^11,13,14,25,34,35^. In addition to attention, theta-band rhythm is also known to be involved in other cognitive functions that deal with multiple items simultaneously. For example, it was found that sustained theta-band power during the delay period in working memory tasks^36^ could predict subsequent retrieval performance^37^. By phase-modulating the gamma-band power, the theta-band rhythm served a critical function in disambiguating individual items held in working memory^38^ and encoding their relationships^39^. Therefore, theta-band rhythm might act as a functional bridge that connects a cascade of cognitive processes^40^, including information selection, representation, storage, and readout, with external behaviors. Functionally, this ubiquitous temporal code is utilized to facilitate the information exchange and coordination between different brain regions that are involved in these cognitive processes^41^, and helps to enhance processing capacity in attention and memory^42,43^.

Previous studies have shown that when a single feature is attended, tuning curves of individual neurons were shifted towards the attended feature^8,26^, thus consistent with the TS model. Interestingly, our modeling results instead support the TC model over the TS model. One possible explanation is that the two mechanisms are optimal under different circumstances. Tuning shift could maximize the sensitivity to the attended feature^4^ during uni-feature attention but would be suboptimal for multi-feature attention because it would be highly inefficient to shift the tunings of almost all neuronal populations every hundreds of milliseconds. In contrast, tuning competition would be more advantageous in implementing the rhythmic sampling process, as it relies on concurrent tuning changes of particular neuronal populations without necessarily involving other neuronal populations^44^. Importantly, we identified rhythmic sharpening and widening of the tuning curves in our modeling at the same theta-band frequency as in the temporal dynamics of the feature representation and behavioral performance. This observation echoes previous findings of attention-induced selectivity increase in visual cortical neurons representing the attended object^45^ or lower-level features^46^. Feature information encoded in neuronal responses is critically constrained by the slope of neuronal tuning curves. Sharpening of a tuning curve increases its slope, which in turn improves the quality of neuronal code and the behavioral discrimination performance^47^. While our modeling results were consistent with previous findings that directing attention to basic visual features, such as color and motion, influences the selectivity of individual neurons^48–51^, given the limited spatial resolution of MEG technique, we cannot assert that the tuning competition occurs at the single-neuron level. It could also occur at the population response level. In fact, tuning width changes at the population level do not necessarily arise from tuning width (or selectivity) changes at the single-neuron level, since a population tuning curve could be affected by changes in amplitudes (or gains) of individual neuronal tuning curves without necessarily changing the shapes (or widths) of the tuning curves^49^.

Although our results are based on neuromagnetic signals from occipital sensors, it is unlikely that our findings only reflect localized neuronal processing in visual cortex. Feature-based attention is known to recruit a large-scale network of brain regions, including V4, frontal eye field (FEF) and ventral prearcuate gyrus (VPA)^3,52^. In specific, it was found that feature-based attention signals arise earlier in VPA than V4, suggesting that the source of feature selection signals might be located outside the visual system^3^. Hence, the rhythmic attentional sampling in the feature space might reflect a dynamic interplay between occipital cortex and higher-order attention-related regions^34,35^. In different phases of a theta-band cycle, top-down signals bias the local competition between feature-selective neurons in visual cortex towards one feature over the other. Consequently, local neuronal computations are temporally orchestrated by higher-order regions, achieving a time-multiplexing representation.

It has recently been proposed that IEM outputs could not be used to infer changes in tuning properties at the single-neuron level, as the IEM outputs were only determined up to a linear transform and thus the recovered model responses are only one of an infinite family of equivalent solutions^53^. However, we believe that these arguments do not speak against the validity of our approach or our findings. While the IEM approach might not be able to provide insights into the tuning properties at the single-neuron level, it does provide an effective way to assay neural representations at the neuronal population level^54^, which enabled us to reveal the rhythmic changes in orientation representations in theta-band. It should be emphasized that here we did not seek to establish a direct link between single-neuron level tuning and population-level representation based on our current findings, though this issue should be investigated in the future.

In summary, we found, for the first time, that multi-feature attention is mediated by a rhythm-based, time-multiplexing neural machinery that sampled each attended feature periodically at theta band. Moreover, the rhythmicity might be implemented by a tuning competition process between the neuronal populations selective to the attended features.

## Methods

### Participants

A total of 30 students (13 males, 18–25 years old) were recruited from Peking University. Fifteen of them (7 males) participated in the MEG experiment and the rest participated in the behavioral experiment. All subjects were naive to the purpose of the study. They were right-handed, reported normal or corrected-to-normal vision, and had no known neurological or visual disorders. Written informed consents were collected from them before the experiments. Experimental procedures were approved by the human subject review committee at Peking University.

### Stimuli and task

The MEG experiment had two parts—the model training part and the attention part. In the model training part, the visual stimulus was an annulus of sinusoidal grating (inner radius = 1.5°, outer radius = 12°, spatial frequency = 0.225 cycles per degree, Michelson contrast = 1, mean luminance = 80 cd/m^2^) centered at fixation. Its phase was randomized and its orientation could be one of six possible orientations from 15° to 165° in steps of 30°. In each trial, the grating stimulus was displayed for 1000–1250 ms, and was then slightly rotated for 150 ms. Subjects indicated the direction of the rotation (clockwise or counterclockwise) (Fig. 1a).

In the attention part, the stimulus consisted of two arrays of phase-randomized sinusoidal gratings (radius = 0.3°, spatial frequency = 3.8 cycles per degree, Michelson contrast = 0.8) oriented at ~45° and ~135°, which were presented in an invisible 9 × 9 rectangular grid centered at fixation. Gratings in the same array shared identical orientation. The size of the grid was 12° × 12°. Spatial randomization of the gratings proceeded in two steps. In the first step, 28 grid cells were randomly selected as the candidate locations for the gratings. In the second step, the location of each grating center was further jittered within the grid cell. The aim of the two-step manipulation was to conceal the spatial configuration of the grid and to discourage subjects from predicting the locations of the gratings, such that subjects’ attention would be directed to both orientation features that were spatially interleaved throughout the grid. Similar to Herrmann et al.^18^, the nine grid cells closest to fixation were intentionally left out such that subjects needed to distribute their attention throughout the grid instead of biasing towards the innermost locations. Each trial started with a 300 ms fixation point. Then the two grating arrays were presented for a random duration between 500 and 1000 ms to minimize potential expectation effects of an upcoming resetting cue. The resetting cue was a 200 ms contrast increment (Δ = 0.2), which could presumably reset the attentional process such that we could examine the moment-by-moment neural representations of the orientation features with reference to a common temporal marker. Similar resetting cues have been employed in previous studies to investigate the temporal dynamics in multi-target attention^10,12–14^. It is important to note that the contrast increment occurred with all gratings in one array rather than in a discrete location. After resetting, the two grating arrays remained on the screen for 1000–1250 ms before they were replaced by a 100 ms probe comprising the same number of gratings oriented at approximately either 45° (right tilted probe) or 135° (left tilted probe). Subjects were fully aware of the cue validity (50%) before the experiment, i.e., in half of the trials the probe orientation was approximately parallel to the cued orientation while in the other trials the probe orientation was approximately perpendicular to the cued orientation. Gratings in the probe went through the same two-step spatial randomization process as described above to minimize location-based selection effects.

Subjects indicated whether the probe orientation was clockwise or counterclockwise of its closest orientation in the two grating arrays. To prevent subjects from holding a reference of the two orientations in memory and using the reference for the task, we randomly introduced a small orientation jitter (±3°) in some trials as in Herrmann et al.^18^ such that the observers were uninformative as to which orientations (42°/138°, 45°/135°, or 48°/132°) would be presented in a trial. Hence, subjects had to attend to the two orientations in each trial in order to complete the task. Inter-trial interval varied randomly between 1000 and 1500 ms. Subjects underwent a total of five blocks for the model training part and eight blocks for the attention part in an interleaved order. Each model training block contained 72 trials (12 for each of the 6 orientations) and each attention block contained 60 trials (30 for each probe condition). Trial order was randomized in all blocks. A two-up-one-down staircase procedure was used in each block to match task difficulty across subjects.

### MEG signal acquisition

Neuromagnetic signals were recorded continuously with a 306-channel (204 planar gradiometers, 102 magnetometers), whole-head MEG system (Elekta Neuromag TRIUX) in a magnetically shielded room. Raw MEG data were offline band-pass filtered between 0.1 and 35 Hz, resampled to 250 Hz, and baseline corrected using Fieldtrip^55^ before subsequent data analysis.

### Optimal orientation pattern localization

For each subject, time-resolved orientation decoding analysis was conducted on the data from the model training part using linear supporting vector machine (libSVM) to localize the optimal orientation pattern. The optimal orientation pattern was defined as the MEG signals that contained the largest amount of orientation information, i.e., the trial-wise vectors of sensor responses that achieved the highest decoding accuracy. Seventy-two sensors covering the occipital lobe that were labeled as Occipital in the MEG data acquisition system were selected for data analyses^24^. Because orientation selectivity is relatively weaker in higher-order brain regions^56^, inclusion of signals from these regions might introduce additional noise. For each time point (from 250 ms before to 1250 ms after stimulus onset), MEG data were arranged in the form of 360 × 72 matrix comprising 360 trial-wise vectors of sensor signals (60 for each orientation category). A six-way decoder was trained to classify these vectors into one of six orientation categories using a leave-one-block-out cross-validation procedure, yielding a decoding accuracy time course for each subject. We extracted the trial-wise vectors of instantaneous sensor signals at the time point with the highest decoding accuracy, which formed the optimal orientation pattern (Fig. 1d). This pattern was then used to train the IEM.

### Reconstruction of the time-resolved orientation representation

According to the assumptions of IEM, instantaneous sensor responses at a single time point across trials could be expressed as a linear combination of the responses of six orientation channels (from 15° to 165° in steps of 30°):

1 $${\mathbf{B}} = {\mathbf{WC}},$$

where **B** is the matrix of sensor signals at a given time point (72 sensors-by-*N* trials), **W** is the matrix of linear weights for the orientation channels (72 sensors-by-6 channels), and **C** is the matrix of channel responses (6 channels-by-*N* trials). The IEM analysis involved two stages: model training and model-based reconstruction. In the model training stage, the mapping from the MEG sensor signals to the six orientation channel outputs (i.e.,**W**) was estimated using the optimal orientation pattern that carried the richest orientation information. To this end, we modeled the idealized tuning in each orientation channel as the half-sinusoidal function raised to the fourth power peaked at the channel’s preferred orientation^57^ (Fig. 1b). Hence, for each trial in the model training sessions, the channel responses could be predicted from these idealized tuning functions (Fig. 1c). Based on the predicted channel responses, the weight matrix **W** could be estimated as

2 $${\hat{\mathbf{W}}} = {\mathbf{B}}_1{\mathbf{C}}_1^{\mathrm{T}}\left( {{\mathbf{C}}_1{\mathbf{C}}_1^{\mathrm{T}}} \right)^{ - 1},$$

where **B**_1_ (72 sensors-by-360 trials) is the optimal orientation pattern matrix obtained as described in the previous section, and **C**_1_ (6 channels-by-360 trials) is the matrix of predicted channel responses for the presented orientation in each trial, which was obtained from the idealized channel tuning functions. It is important to note that estimation of **W** involved only the model training part data and that the mapping was assumed to be invariant across the model training part data and the attention part data. In the model-based reconstruction stage, the weight matrix was then applied to the instantaneous sensor signals in the attention part data (i.e., **B**_2_) at each time point to estimate the instantaneous individual channel responses:

3 $${\mathbf{C}}_2 = \left( {{\hat{\mathbf{W}}}^{\mathrm{T}}{\hat{\mathbf{W}}}} \right)^{ - 1}{\hat{\mathbf{W}}}^{\mathrm{T}}{\mathbf{B}}_2,$$

where **B**_2_ (72 sensors-by-480 trials) is the matrix of instantaneous sensor signals in the attention part data and **C**_2_ (6 channels-by-480 trials) is the matrix comprising column vectors of estimated trial-wise channel responses. These trial-wise vectors were then averaged, yielding one vector of channel responses for each time point (Fig. 1e). Hence, the orientation information in each trial is represented in the channel space. To quantify to what extent the channel responses encode the information of a given orientation, we computed the representational fidelity metric as a mean of six unit vectors pointing in the polar angle directions corresponding to the channels’ preferred orientations and weighted by the estimated channel responses^21^

4 $${\vec{\mathbf{z}}} = \frac{{\mathop {\sum }\nolimits_k {\mathrm{c}}_k{\vec{\mathbf{u}}}_k}}{6},k = 1,2, \ldots ,6,$$

where ${\vec{\mathbf{z}}}$ is the resultant mean vector, c_*k*_ is the response of the *k*th channel, and ${\vec{\mathbf{u}}}_k$ is its corresponding unit vector. This mean vector was then projected onto a unit vector pointing in the polar angle direction along the orientation of interest (the six orientations in the model training part, 45° and 135° in the attention part),

5 $${{R}} = \left| {{\vec{\mathbf{z}}}} \right|\cos \phi,$$

where *ϕ* is the angle between the mean vector and the polar angle direction along the orientation of interest. The resultant value *R* hence provide a quantitative measure of the extent to which the information of a given orientation was encoded in the channel responses, with a greater *R* value above zero indicating a stronger representation of the orientation of interest (Fig. 1f). Using this approach, we calculated the representation fidelity of the orientation of interest at each time point (Fig. 1g), which yielded the orientation representation fidelity time course. For the attention part data, we epoched the representation fidelity time courses from 0 to 1000 ms after the onset of the resetting cue, removed the linear trends of these epochs and performed spectral analysis using Fast Fourier Transform (FFT). Amplitude and phase spectra were derived by taking the absolute value and the phase value of the complex Fourier coefficients, respectively. The subject-wise amplitude spectra were averaged to obtain the group-level results.

### Statistical procedures

Statistical significance of the amplitude spectrum of the representation fidelity time course was assessed using a permutation procedure. In each iteration, we performed FFT on the original time course, randomly shuffled the phase spectrum and performed inverse FFT on the phase-scrambled representation fidelity time course to produce the surrogate signal. We then performed the same analysis on the surrogate signal to derive the amplitude spectrum. This randomization procedure was repeated 1000 times and yielded a distribution of spectral power at each frequency bin, from which the corresponding thresholds (*p* *=* 0.05, uncorrected) were obtained. To correct for multiple comparisons, we used a cross-frequency correction approach^14^, i.e., the maximum threshold value across all frequency bins was set as the threshold. Notably, the randomization procedure was performed separately for each individual subject, which took the cross-subject variance into account. Statistical assessment of phase relations was conducted using CircStat Toolbox^58^. For each subject, the phase difference between the two orientation representation fidelity time courses at the peak frequency was computed and tested using Rayleigh’s test for non-uniformity.

### Time-resolved psychophysics

The time-resolved psychophysical experiment was performed in a behavioral test room. The visual stimuli and task procedure were identical to those in the attention part of the MEG experiment with one critical exception. Namely, we systematically manipulated the cue-to-probe stimulus onset asynchrony (SOA, 50–1050 ms) in steps of 20 ms instead of using a random interval (1–1.25 s), which corresponded to a sampling rate of 50 Hz in the behavioral time course. The visual stimuli were presented on an IIYAMA HM204DT 22-inch monitor with a spatial resolution of 1024 × 768. Subjects viewed the stimuli from a distance of 70 cm with their heads stabilized on a chin rest. The behavioral experiment consisted of 40 blocks. Each block had 50 trials with one trial per SOA. For each SOA, in half of the trials, the probe orientation was ~45° ± *θ*° while in the other half the probe orientation was ~135° ± *θ*°. Prior to the experiment, the orientation discrimination threshold *θ* was independently determined for individual subjects in a pilot experiment using the same stimuli and task to match task difficulty across subjects. Similar to the attention part of the MEG experiment, the validity of the perceptual cue was 50%. For both attended orientations, we derived the behavioral time course by measuring orientation discrimination accuracy as a function of SOA. Analyses and statistical assessment of the amplitude spectra and the phase relationship of the Fourier components at the peak frequency were performed using the same procedure as described in the previous sections.

### Model fitting and comparison

Prior to model fitting, we first generated the smooth population response profile within 0–180° at each time point by summing the idealized channel tuning functions weighted by their estimated responses^21^. We then fitted the two candidate models (the tuning shift model and the tuning competition model) to the population response profile at each time point for each subject. For the tuning shift model, we used an exponentiated cosine function:^19^

6 $$f\left( {x|A,K,\mu ,b} \right) = Ae^{K\{ {\mathrm{cos}}[\left( {\mu - x} \right)] - 1\} } + b,$$

where *x* is the orientation value, *A*, *K*, and *b* are the amplitude, concentration, and baseline of the function. The critical model parameter *μ* determines the peak orientation of the population response profile, which, according to the model hypothesis, would rhythmically shift between 45° and 135°. The tuning competition model was defined as the weighted sum of two exponentiated cosine functions that characterized the tuning of the neuronal populations selective to the two attended orientations (i.e., 45° and 135°):

7 $$\begin{array}{l}f\left( {x|A_1,K_1,A_2,K_2,b} \right) = A_1e^{K_1\{ {\mathrm{cos}}[\left( {\frac{\pi }{4} - x} \right)] - 1\} } + A_2e^{K_2\{ {\mathrm{cos}}[\left( {\frac{{3\pi }}{4} - x} \right)] - 1\} } + b\end{array},$$

where *A*_1_, *K*_1_, *A*_2_, and *K*_2_ denote the amplitude and the concentration of the two tuning curves. For both models, parameters were varied to obtain the minimal sum of squared errors between the population response profile and the model prediction. To statistically compare the two models, we computed the root mean squared deviation (RMSD) of the two fitted models for each time point:

8 $${\mathrm{RMSD}} = \sqrt {{\mathrm{SSE}}/\left( {N - k} \right)},$$

where SSE is the sum of squared errors. *N* is the number of data points (i.e., 180), and *k* is the number of model parameters. For each subject and each model, we calculated the mean RMSD by averaging the model’s RMSD values across all time points. A non-parametric Wilcoxon’s signed rank test was conducted to compare the RMSD values of the two models across subjects.

### Reporting Summary

Further information on research design is available in the Nature Research Reporting Summary linked to this article.

## Supplementary information

Supplementary Information

Reporting Summary

## Data Availability

Data supporting the findings of this study are available from the corresponding authors upon request.

## Code availability

Codes associated with the findings of this study are available from the corresponding authors upon request.
