# Supplementary material for: Competing rhythmic neural representations of orientations during concurrent attention to multiple orientation features
Source: Nat Commun. 2019 Nov 20;10:5264. doi: 10.1038/s41467-019-13282-3 (PMC6868242; doi:10.1038/s41467-019-13282-3)
Supplement: Supplementary file 1 — Supplementary Information [file 41467_2019_13282_MOESM1_ESM.pdf]

Competing rhythmic neural representations of orientations during concurrent attention to multiple orientation features

Mo et al.

Supplementary figures

Supplementary Figure 1

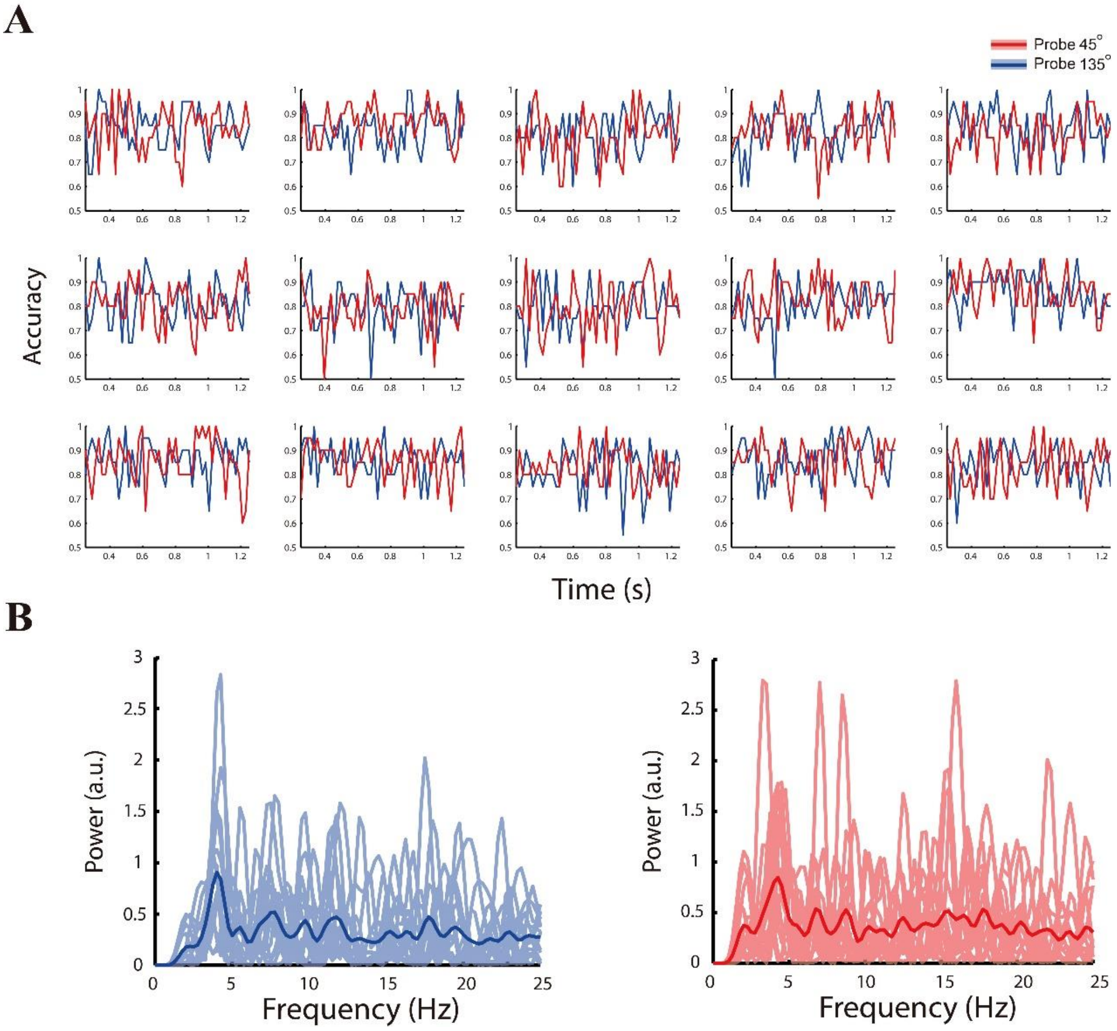

Supplementary Figure 1. Behavioral results for individual subjects

A) Raw time-resolved behavioral courses for individual subjects. B) Power spectra for individual subjects are shown in desaturated colors. Group averaged results are shown in saturated colors.

Supplementary Figure 2

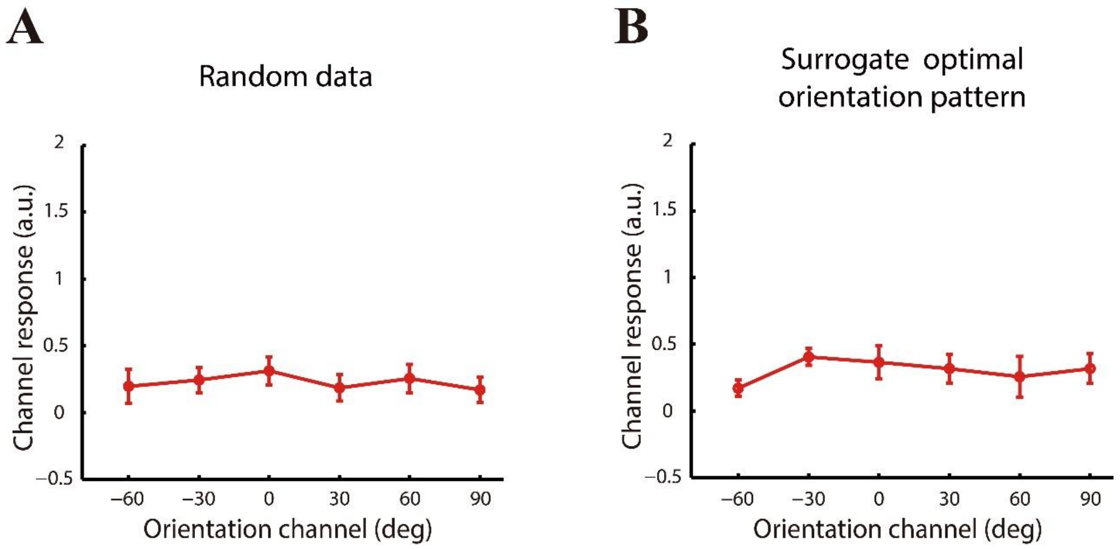

Supplementary Figure 2. Validation of the IEM approach.

Channel response functions in the post-stimulus period obtained by applying the same IEM model to the surrogate data (Left) and by applying the IEM model trained by the surrogate optimal orientation pattern (i.e., the trial-wise pattern of instantaneous sensor signals that yielded the highest orientation decoding performance in the pre-stimulus period) to the original data (Right). Neither of them exhibited a bell-shaped profile that suggests representation of orientation information.
