# Supplementary material for: Competing rhythmic neural representations of orientations during concurrent attention to multiple orientation features
Source: Nat Commun. 2019 Nov 20;10:5264. doi: 10.1038/s41467-019-13282-3 (PMC6868242; doi:10.1038/s41467-019-13282-3)
Supplement: Supplementary file 2 — Reporting Summary [file 41467_2019_13282_MOESM2_ESM.pdf]

nature research

Corresponding author(s): Fang Fang, Huan Luo

Last updated by author(s): Oct 30, 2019

Reporting Summary

Nature Research wishes to improve the reproducibility of the work that we publish. This form provides structure for consistency and transparency in reporting. For further information on Nature Research policies, see Authors & Referees and the Editorial Policy Checklist.

Statistics

For all statistical analyses, confirm that the following items are present in the figure legend, table legend, main text, or Methods section.

n/a	Confirmed	
☐	☒	The exact sample size (n) for each experimental group/condition, given as a discrete number and unit of measurement
☐	☒	A statement on whether measurements were taken from distinct samples or whether the same sample was measured repeatedly
☐	☒	The statistical test(s) used AND whether they are one- or two-sided<br>*Only common tests should be described solely by name; describe more complex techniques in the Methods section.*
☒	☐	A description of all covariates tested
☐	☒	A description of any assumptions or corrections, such as tests of normality and adjustment for multiple comparisons
☐	☒	A full description of the statistical parameters including central tendency (e.g. means) or other basic estimates (e.g. regression coefficient) AND variation (e.g. standard deviation) or associated estimates of uncertainty (e.g. confidence intervals)
☐	☒	For null hypothesis testing, the test statistic (e.g. F, t, r) with confidence intervals, effect sizes, degrees of freedom and P value noted<br>*Give P values as exact values whenever suitable.*
☒	☐	For Bayesian analysis, information on the choice of priors and Markov chain Monte Carlo settings
☒	☐	For hierarchical and complex designs, identification of the appropriate level for tests and full reporting of outcomes
☒	☐	Estimates of effect sizes (e.g. Cohen's d, Pearson's r), indicating how they were calculated

Our web collection on statistics for biologists contains articles on many of the points above.

Software and code

Policy information about availability of computer code

Data collection	Behavioral data was collected using Psychtoolbox 3 based on Matlab R2014a (Mathworks, Natick, MA). MEG data was collected using data acquisition software suite with Elekta Neuromag TRIUX (Elekta, Stockholm, Sweden).
Data analysis	Data were analyzed using Fieldtrip toolbox and custom scripts based on Matlab 2014a.

For manuscripts utilizing custom algorithms or software that are central to the research but not yet described in published literature, software must be made available to editors/reviewers. We strongly encourage code deposition in a community repository (e.g. GitHub). See the Nature Research guidelines for submitting code & software for further information.

Data

Policy information about availability of data

All manuscripts must include a data availability statement. This statement should provide the following information, where applicable:

- Accession codes, unique identifiers, or web links for publicly available datasets
- A list of figures that have associated raw data
- A description of any restrictions on data availability

The data supporting the findings of this study are available from the corresponding author upon reasonable request.

Field-specific reporting

Please select the one below that is the best fit for your research. If you are not sure, read the appropriate sections before making your selection.

☒ Life sciences ☐ Behavioural & social sciences ☐ Ecological, evolutionary & environmental sciences

October 2018

Life sciences study design

All studies must disclose on these points even when the disclosure is negative.

Sample size	The sample size was determined based on the reported sample sizes in previous related studies. Independent pilot studies were conducted to ensure that the chosen sample size was sufficient.
Data exclusions	No data was excluded from the analyses.
Replication	After the collection of all data, data analyses were first done with half of the subject's data and then re-done with the other half, which yielded consistent findings as reported.
Randomization	The experimental design did not involve allocation of participants into different groups.
Blinding	Blinding was not relevant for the study, as it did not involve group allocation.

Reporting for specific materials, systems and methods

We require information from authors about some types of materials, experimental systems and methods used in many studies. Here, indicate whether each material, system or method listed is relevant to your study. If you are not sure if a list item applies to your research, read the appropriate section before selecting a response.

Materials & experimental systems

n/a	Involved in the study	
☒	☐	Antibodies
☒	☐	Eukaryotic cell lines
☒	☐	Palaeontology
☒	☐	Animals and other organisms
☐	☒	Human research participants
☒	☐	Clinical data

Methods

n/a	Involved in the study	
☒	☐	ChIP-seq
☒	☐	Flow cytometry
☒	☐	MRI-based neuroimaging

Human research participants

Policy information about studies involving human research participants

Population characteristics	Participants were 30 healthy undergraduate students (13 male, 18-25 years old) from Peking University. All participants were right-handed, reported normal or corrected-to-normal vision, and had no history of known neurological or visual disorders.
Recruitment	Participants were volunteers publicly recruited via the official bulletin board system (BBS) of Peking University, who received monetary payment for their participation. Since all participants and were randomly selected from the signed-up candidates, we do not expect any selection biases that might impact the results. All participants were fully aware of the experimental protocol before the experiment.
Ethics oversight	Experimental procedures were approved by the human subject review committee at Peking University.

Note that full information on the approval of the study protocol must also be provided in the manuscript.
